# Room-temperature deformation of single crystals of ZrB_2_ and TiB_2_ with the hexagonal AlB_2_ structure investigated by micropillar compression

**DOI:** 10.1038/s41598-021-93693-9

**Published:** 2021-07-12

**Authors:** Zhenghao Chen, Bhaskar Paul, Sanjib Majumdar, Norihiko L. Okamoto, Kyosuke Kishida, Haruyuki Inui, Shigeki Otani

**Affiliations:** 1 Center for Elements Strategy Initiative for Structure Materials (ESISM), Kyoto University, Sakyo-ku, Kyoto, 606-8501 Japan; 2 Department of Materials Science and Engineering, Kyoto University, Sakyo-ku, Kyoto, 606-8501 Japan; 3 High Temperature Materials Development Section, Materials Processing and Corrosion Engineering Division, Bhabha Atomic Research Centre, Trombay, Mumbai, 400085 India; 4 Institute for Materials Research, Tohoku University, Aoba-ku, Sendai, Miyagi 980-8577 Japan; 5 PRESTO, Japan Science and Technology Agency, 4-1-8 Honcho, Kawaguchi, Saitama 332-0012 Japan; 6 Research Center for Functional Materials, National Institute for Materials Science, 1-1, Namiki, Tsukuba, Ibaraki 305-0044 Japan

**Keywords:** Ceramics, Mechanical properties

## Abstract

The plastic deformation behavior of single crystals of two transition-metal diborides, ZrB_2_ and TiB_2_ with the AlB_2_ structure has been investigated at room temperature as a function of crystal orientation and specimen size by micropillar compression tests. Although plastic flow is not observed at all for their bulk single crystals at room temperature, plastic flow is successfully observed at room temperature by the operation of slip on {1${\bar{1}}$00}<11${\bar{2}}$3> in ZrB_2_ and by the operation of slip on {1${\bar{1}}$00}<0001> and {1${\bar{1}}$00}<11${\bar{2}}$0> in TiB_2_. Critical resolve shear stress values at room temperature are very high, exceeding 1 GPa for all observed slip systems; 3.01 GPa for {1${\bar{1}}$00}<11${\bar{2}}$3> slip in ZrB_2_ and 1.72 GPa and 5.17 GPa, respectively for {1${\bar{1}}$00}<0001> and {1${\bar{1}}$00}<11${\bar{2}}$0> slip in TiB_2_. The identified operative slip systems and their CRSS values are discussed in comparison with those identified in the corresponding bulk single crystals at high temperatures and those inferred from micro-hardness anisotropy in the early studies.

## Introduction

Transition-metal diborides, ZrB_2_ and TiB_2_ with the hexagonal AlB_2_ structure (Pearson symbol: *hP*3, space group: *P*6/*mmm*) are important members of ultra-high temperature ceramics (UHTC)^1–7^, because of their numerous unique and advantageous physical and chemical properties required for ultra-high temperature applications^8–10^. Many transition-metal diborides including the above diborides have been considered to be promising candidate materials for a number of applications such as cutting tool^11,12^, armor^11,12^, diffusion barrier for preventing electromigration^13^, electrodes^14^, refractory structural materials for hypersonic vehicles^15–17^, control rod for nuclear reactors^11^ and so on^8–10^. Owing to the technological importance, mechanical properties of transition-metal diborides have been investigated for a several decades^18–24^. Many of studies in the early days utilized micro-hardness anisotropy to infer operative slip systems at room temperature, although only slip along <11$\bar{2}$0> directions (***a*** slip) on either basal or prism plane was considered^18–20^. However, the operative slip systems at room temperature thus inferred for transition-metal diborides are somewhat controversial. For example, in ZrB_2_ single crystals, the operation of {1$\bar{1}$00}<11$\bar{2}$0> (prism ***a***) slip was inferred by Haggerty et al.^20^, while Nakano et al.^19^ inferred the operation of (0001)<11$\bar{2}$0> (basal ***a***) slip. Similarly, in TiB_2_ single crystals, the operation of both {1$\bar{1}$00}<11$\bar{2}$0> and (0001)<11$\bar{2}$0> slip was inferred by Mersol et al.^18^, whereas Nakano et al.^19^ inferred the operation of only {1$\bar{1}$00}<11$\bar{2}$0> slip. Later, slip involving the *c*-axis component has been claimed to participate in deformation of transition-metal diborides at room temperature^21,22^. Ghosh et al.^21^ identified the operation of {1$\bar{1}$00}[0001] (prism ***c***) slip by transmission electron microscopy (TEM) in scratch-induced groove by nano-indentation on a ZrB_2_-SiC composite. Csanadi et al.^22^ then inferred the operation of {1$\bar{1}$00}[0001] and {1$\bar{1}$00}<11$\bar{2}$3> slip at room temperature in ZrB_2_ from slip patterns around cube corner of nano-indent. In contrast to the intensive studies with indentation methods to infer operative slip systems at room temperature, the number of studies to investigate the macroscopic flow behavior of transition-metal diborides with compression/tensile tests of bulk single crystals is very much limited^20,23,24^. Haggerty et al.^20^ observed plastic flow of ZrB_2_ single crystals occurring only above 2050 °C in compression by the operation of (0001)<11$\bar{2}$0> slip, while Ramberg et al.^23^ observed plastic flow in TiB_2_ polycrystals occurring only above 1700 °C in compression without identifying the operative slip systems. However, these studies are far from systematic to understand the macroscopic flow behavior of transition-metal diborides since orientation- and temperature-dependent operative slip systems and their critical resolved shear stress (CRSS) values are not elucidated at all. Recently, we have made a first systematic study of the plastic deformation behavior of single crystals of TiB_2_ and ZrB_2_ in compression as a function of crystal orientation and temperature in a wide range from room temperature to 1500 °C^25^. In ZrB_2_, plastic flow is observed above 700 °C by the operation of slip on {1$\bar{1}$00}<11$\bar{2}$3> (prism ***a*** + ***c***) and above 800 °C by the operation of slip on (0001)<11$\bar{2}$0> (basal ***a***), whereas any appreciable plastic flow is not observed in TiB_2_ below 1500 °C. The absence of plastic flow below 1500 °C in TiB_2_ is considered to be closely associated with the existence of high density of grown-in stacking faults on prism planes. Of particular importance to notice is that plastic flow is not observed at room temperature in bulk single crystals of both transition-metal diborides. This clearly indicates that plastic flow observed at room temperature in the vicinity of micro-hardness indents in transition-metal diborides in the early studies^18–22^ is due to small-scale plasticity that does not occur in bulk but often occurs in small volume of brittle material even at temperatures well below the brittle-ductile transition temperature^26–31^. Micropillar compression testing that utilizes specimen of micrometer size^32–34^ has increasingly been known in recent years to offer one of the best ways to investigate such small-scale plasticity of brittle materials with many accumulated examples^35–43^. Indeed, Csanadi et al.^44^ recently utilized micropillar compression testing and confirmed plastic flow occurring by the operation of {1$\bar{1}$00}<11$\bar{2}$3> (prism ***a*** + ***c***) slip in ZrB_2_ at room temperature. Interestingly, the slip system they identified in micropillar testing at room temperature is completely different from those ({1$\bar{1}$00}<11$\bar{2}$0>^20^ and (0001)<11$\bar{2}$0>^19^) inferred from micro-hardness anisotropy but coincides with one of the two slip systems we identified to operate in bulk single crystals above 700 °C^25^. However, since crystal orientations they tested in micropillar compression (two orientations near [0001] and [1$\bar{1}$00]) are not favored to activate slip systems other than {1$\bar{1}$00}<11$\bar{2}$3> (prism ***a*** + ***c***) slip, whether or not another slip system ((0001)< 11$\bar{2}$0>) we identified to operate in bulk ZrB_2_ single crystals at high temperatures can operate at room temperature is not clear. On top of that, whether or not TiB_2_ also exhibits plastic flow at room temperature in micropillar compression and if so, which slip systems are activated in TiB_2_ are not clear at all yet.

In the present study, we investigate the plastic deformation behavior of single crystals of TiB_2_ and ZrB_2_ at room temperature as a function of crystal orientation and specimen size by micropillar compression testing, in order to see if plastic flow occurs in these transition-metal diborides and to identify the operative slip systems and their CRSS values. We also pay attention to the different *c*/*a* axial ratios for ZrB_2_ (1.123) and TiB_2_ (1.066) to see if the *c*/*a* axial ratio affects the operative slip systems. We discuss the identified operative slip systems and their CRSS values in comparison with those identified in the corresponding bulk single crystals at high temperatures^25^ and with those inferred from micro-hardness anisotropy in the early studies^18–20^.

## Results

### Stress–strain behavior and size-dependent strength

**Figure 1:**
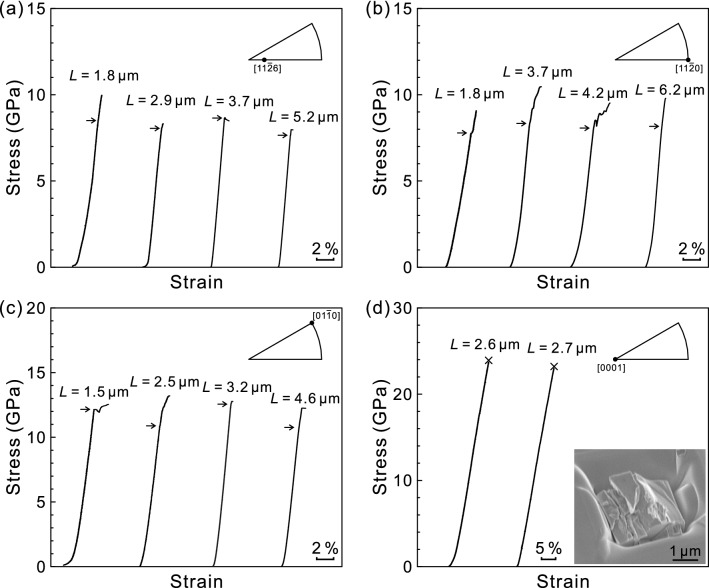
Selected stress–strain curves obtained for micropillar specimens of ZrB_2_ with (**a**) [11$\bar{2}$6], (**b**) [11$\bar{2}$0], (**c**) [01$\bar{1}$0] and (**d**) [0001] orientations. Yield stresses are defined either as the elastic limit at which the stress–strain behavior deviates from the linear relationship or as the stress at which the first strain burst occurs, as indicated by arrows. The inset figure of (**d**) corresponds to the [0001]-oriented specimen with *L* = 2.7 μm.

Selected stress–strain curves for single-crystal micropillar specimens of ZrB_2_ with the [11$\bar{2}$6], [11$\bar{2}$0], [01$\bar{1}$0] and [0001] orientations are shown in Fig. 1a–d, respectively. The specimen sizes (edge length *L*) tested are indicated for each of stress–strain curves. Compression tests were usually intended to interrupt before failure occurs for the ease of slip trace observations. However, failure occurred often prematurely or immediately after yielding for many cases of [11$\bar{2}$6], [11$\bar{2}$0] and [01$\bar{1}$0] orientations. Stress–strain curves shown in Fig. 1a–c correspond thus to only those specimens in which yielding was successfully observed. On the other hand, premature failure always occurred at the stress level exceeding 20 GPa without exhibiting any appreciable plastic strain prior to failure for micropillars with the [0001] orientation (see the inset figure of specimen after premature failure in Fig. 1d). The occurrence of premature failure at a very high stress level exceeding 20 GPa for the [0001] orientation of ZrB_2_ is consistent with the result of micropillar compression by Csanadi et al.^44^. For the other three orientations, yielding is defined either as the elastic limit at which the stress–strain behavior deviates from the linear relationship or as the stress at which the first strain burst occurs, as indicated by arrows in Fig. 1a–c. The magnitude of strain (flat portions of stress–strain curves) for each burst as well as the occurrence of strain burst itself depends on individual specimen without any definite trend in terms of specimen size and stress level for all the three orientations of Fig. 1a–c. The magnitude of strain for each burst in ZrB_2_ seems to be rather small when compared to many other brittle materials such as SiC^39^ and Mo_5_SiB_2_^37^. The yield stress thus defined for all the three orientations ([11$\bar{2}$6], [11$\bar{2}$0] and [01$\bar{1}$0]) of ZrB_2_ seems not to depend much on the specimen size (edge length *L*), although the experimental scatter is very large to safely conclude.

**Figure 2:**
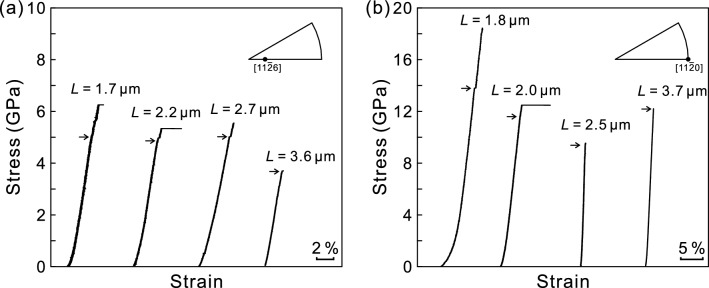
Selected stress–strain curves obtained for micropillar specimens of TiB_2_ with (**a**) [11$\bar{2}$6] and (**b**) [11$\bar{2}$0] orientations. Yield stresses are defined either as the elastic limit at which the stress–strain behavior deviates from the linear relationship or as the stress at which the first strain burst occurs, as indicated by arrows.

Selected stress–strain curves for single-crystal micropillar specimens of TiB_2_ with the [11$\bar{2}$6], and [11$\bar{2}$0] are shown in Fig. 2a,b, respectively. The stress–strain behaviors of all these specimens are very similar to those observed for micropillar specimens of ZrB_2_ with the [11$\bar{2}$6], [11$\bar{2}$0] and [01$\bar{1}$0] orientations; failure occurred often prematurely or immediately after yielding. As in the case of ZrB_2_, the yield stress is defined either as the elastic limit or as the first strain burst stress, as indicated by arrows in Fig. 2a,b and it is seen not to depend much on the specimen size (edge length L), although the experimental scatter is again very large to safely conclude. Of significance to note is the occurrence of room-temperature plastic flow in TiB_2_, in which any appreciable plastic flow was not observed at all for bulk single crystals below 1500 °C in our previous study^25^.

**Figure 3:**
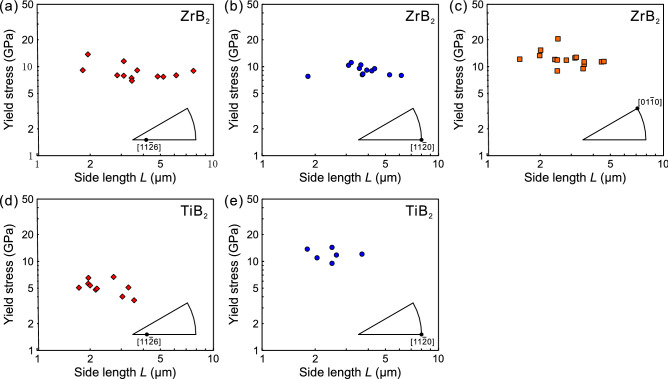
Variation of yield stress with specimen size for (**a**) [11$\bar{2}$6], (**b**) [11$\bar{2}$0], (**c**) [01$\bar{1}$0] orientations of ZrB_2_ and for (**d**) [11$\bar{2}$6] and (**e**) [11$\bar{2}$0] orientations of TiB_2_.

The variation of yield stresses with specimen size (edge length *L*) is plotted in Fig. 3a–c, respectively for [11$\bar{2}$6], [11$\bar{2}$0] and [01$\bar{1}$0] orientations of ZrB_2_ and in Fig. 3d,e, respectively for [11$\bar{2}$6] and [11$\bar{2}$0] orientations of TiB_2_. Although the experimental scatter is again very large to safely conclude, the yield stress seems not to depend much on the specimen size for all cases of Fig. 3a–e. The yield stresses obtained for all three orientations of ZrB_2_ are very high, being distributed around 10 GPa. Although this is also the case for the [11$\bar{2}$0] orientation of TiB_2_, the yield stresses for the [11$\bar{2}$6] orientation of TiB_2_ are a bit smaller, being distributed in the range of 3–7 GPa. Of interest to note in Fig. 3 is that while TiB_2_ is exceptionally brittle in bulk as any plastic flow is not observed at all below 1500 °C^25^, the magnitude of the yield stress at room temperature for TiB_2_ is in the same range (for the [11$\bar{2}$0] orientation) or a bit smaller (for the [11$\bar{2}$6] orientation) than those for ZrB_2_.

### SEM deformation structures (slip system identification)

**Figure 4:**
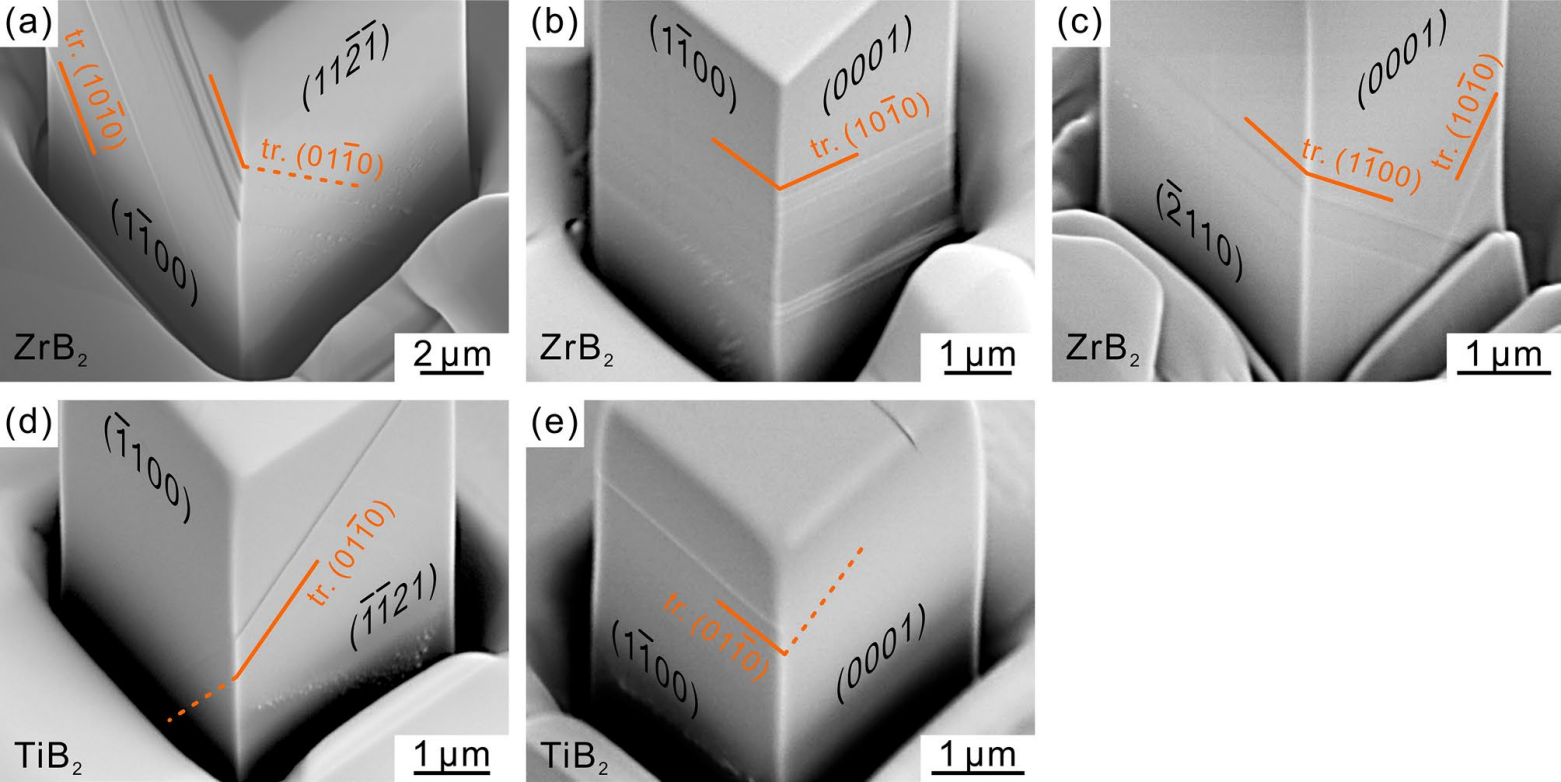
SEM secondary electron images of deformed micropillar specimens taken diagonally from the above (inclined by 30° from the loading axis) for (**a**–**c**) ZrB_2_, (**d**,**e**) TiB_2_ with (**a**,**d**) [11$\bar{2}$6], (**b**,**e**) [11$\bar{2}$0] and (**c**) [01$\bar{1}$0] orientations.

Figure 4a–c show SEM secondary electron images of deformation structures observed for micropillar specimens of ZrB_2_ with the [11$$\bar{2}$$6], [11$$\bar{2}$$0] and [01$$\bar{1}$$0] orientations, respectively. The observations were made along the direction inclined by 30° from the loading axis. For the [11$$\bar{2}$$6] orientation (Fig. 4a), straight slip traces are clearly observed on the (1$$\bar{1}$$00) surface, while slip traces observed on the ($$\bar{1}$$$$\bar{1}$$21) surface are fairly faint. Slip trace analysis on the two orthogonal surfaces indicates the occurrence of slip on (01$$\bar{1}$$0) and (10$$\bar{1}$$0). The faint slip lines on the (11$$\bar{2}$$$$\bar{1}$$) surface indicates that the slip vector is contained in the (11$$\bar{2}$$$$\bar{1}$$) plane. Stereographic analysis has revealed that the slip directions are parallel to [$$\bar{2}$$11$$\bar{3}$$] and [1$$\bar{2}$$1$$\bar{3}$$] on (01$$\bar{1}$$0) and (10$$\bar{1}$$0), respectively. The slip system thus identified to operate in the micropillar with the [11$$\bar{2}$$6] orientation are (01$$\bar{1}$$0)[$$\bar{2}$$11$$\bar{3}$$] and (10$$\bar{1}$$0)[1$$\bar{2}$$1$$\bar{3}$$] (prism ***a*** + ***c***). Although the slip system identified to operate in bulk single crystals of ZrB_2_ with the same orientation at high temperatures above 800 °C is basal ***a*** slip^25^, this is replaced by prism ***a*** + ***c*** slip at room temperature.

For the [11$$\bar{2}$$0] orientation (Fig. 4b), straight slip traces corresponding slip on (10$$\bar{1}$$0) are clearly observed on both the (1$$\bar{1}$$00) and (0001) surfaces. Since there is no resolved shear stress for prism ***c*** slip and basal ***a*** slip, either prism ***a*** slip or prism ***a*** + ***c*** slip should operate for this orientation. The intense slip traces observed on the (0001) surface, on which the ***a*** slip directions are contained, clearly denies the operation of prism ***a*** slip, indicating the operation of prism ***a*** + ***c*** ((10$$\bar{1}$$0)[1$$\bar{2}$$13] and (10$$\bar{1}$$0)[1$$\bar{2}$$1$$\bar{3}$$]) slip for the [11$$\bar{2}$$0] orientation. This slip system coincides with that observed to operate in bulk single crystals of ZrB_2_ with the same orientation at high temperatures above 700 °C^25^. The operative slip system for the [01$$\bar{1}$$0] orientation is similarly determined to be prism ***a*** + ***c*** ((1$$\bar{1}$$00)[11$$\bar{2}$$3], (1$$\bar{1}$$00)[11$$\bar{2}$$$$\bar{3}$$], (10$$\bar{1}$$0)[1$$\bar{2}$$13] and (10$$\bar{1}$$0)[1$$\bar{2}$$1$$\bar{3}$$]) slip from the slip trace analysis of Fig. 4c that straight slip traces corresponding slip on (1$$\bar{1}$$00) and (10$$\bar{1}$$0) are clearly observed on both the ($$\bar{2}$$110) and (0001) surfaces. The operation of prism ***a*** + ***c*** slip for the [01$$\bar{1}$$0] orientation of ZrB_2_ is consistent with the result of micropillar compression by Csanadi et al.^44^. The operative slip system (prism ***a*** + ***c*** slip) identified for each orientation does not vary with specimen size.

Figure 4d,e show SEM secondary electron images of deformation structures observed for micropillar specimens of TiB_2_ with the [11$$\bar{2}$$6] and [11$$\bar{2}$$0] orientations, respectively. The slip system operative for the [11$$\bar{2}$$6] orientation is determined to be (01$$\bar{1}$$0)[0001] (prism ***c***) from the fact that the intense and faint slip traces corresponding to slip on (01$$\bar{1}$$0) are observed respectively for the ($$\bar{1}$$$$\bar{1}$$21) and ($$\bar{1}$$100) surfaces (Fig. 4d). For the [11$$\bar{2}$$0] orientation (Fig. 4e), straight slip traces corresponding slip on (01$$\bar{1}$$0) are clearly observed on the (1$$\bar{1}$$00) surface while they are only faint on the (0001) surface. As described above, the possible slip systems to operate for this orientation are either prism ***a*** slip or prism ***a*** + ***c*** slip. Since the ***a*** slip directions are contained on the (0001) surface on which slip traces are only faintly observed, the operative slip system for this orientation is determined to be prism ***a*** ((01$$\bar{1}$$0)[2$$\bar{1}$$$$\bar{1}$$0]). Although prism ***a*** slip was inferred to operate in TiB_2_ from micro-hardness anisotropy^18,19^, this slip system was not identified to operate in bulk single crystals of any of ZrB_2_, TiB_2_ and CrB_2_ in our previous study^25^.

### Specimen size-dependent CRSS

**Figure 5:**
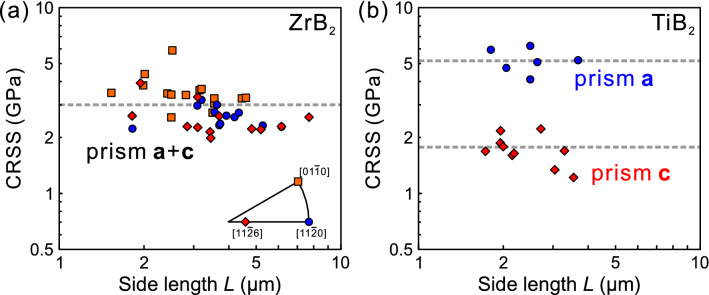
CRSS values for the observed slip systems plotted as a function of specimen size for (**a**) {1$\bar{1}$00}<11$\bar{2}$3> slip in ZrB_2_, (**b**) {1$\bar{1}$00}<0001> and {1$\bar{1}$00}<11$\bar{2}$0> slip in TiB_2_.

The slip systems identified to operate are slip on {1$\bar{1}$00}<11$\bar{2}$3> slip in ZrB_2_ and slip on {1$\bar{1}$00}<0001> and {1$\bar{1}$00}<11$\bar{2}$0> in TiB_2_. CRSS values for these slip systems calculated with the yield stress and the corresponding Schmid factor of the relevant slip system (Table 1) are plotted in Fig. 5a,b as a function of specimen size for ZrB_2_ and TiB_2_. The CRSS values for all these slip systems do not much depend on specimen size, although an inverse power-law scaling (i.e., CRSS ∝ *L*^−*n*^) is established for many conventional metal and alloys^32–34,45–49^. If the average value is taken, the CRSS values are estimated to be 3.01 GPa for {1$\bar{1}$00}<11$\bar{2}$3> slip in ZrB_2_ and 1.72 GPa and 5.17 GPa, respectively for {1$\bar{1}$00}<0001> and {1$\bar{1}$00}<11$\bar{2}$0> slip in TiB_2_.

**Table 1:** The ***c***/***a*** axial ratios and Schmid factors for various slip systems expected to operate depending on compression-axis orientations employed for ZrB_2_ and TiB_2_.

	Orientation	$\{ 1\bar{1}00\} < 11\bar{2}0 >$ slip	$\{ 1\bar{1}00\} [0001]$ slip	$\{ 1\bar{1}00\} < 11\bar{2}6 >$ slip	$(0001) < 11\bar{2}0 >$ slip
ZrB_2_ *c*/*a* = 1.129	[0001]	0	0	0	0
	$[11\bar{2}0]$	0.433	0	0.287	0
	$[01\bar{1}0]$	0.433	0	0.287	0
	$[11\bar{2}6]$	0.071	0.321	0.287	0.370
TiB_2_ *c*/*a* = 1.066	$[11\bar{2}0]$	0.433	0	0.296	0
	$[11\bar{2}6]$	0.078	0.333	0.296	0.384

## Discussion

### Operative slip systems and CRSS

In ZrB_2_, {1$\bar{1}$00}<11$\bar{2}$3> (prism ***a*** + ***c*** slip) slip, which was identified as the primary slip system at high temperatures in bulk single crystals, is only the operative slip system at room temperature. In TiB_2_, on the other hand, {1$\bar{1}$00}<0001> (prism ***c*** slip) and {1$\bar{1}$00}<11$\bar{2}$0> (prism ***a*** slip) slip are observed to operate at room temperature.

In early studies of micro-hardness anisotropy, basal ***a*** slip and prism ***a*** slip are inferred to be the primary slip system in transition-metal diborides, although the argument usually considered the possibility of only these two slip systems ignoring the possibility of any other slip systems containing the ***c***-axis component^18–20^. Basal ***a*** slip was claimed to be the primary system in ZrB_2_ by Nakano et al.^19^ while prism ***a*** slip was claimed so in ZrB_2_ by Haggerty et al.^20^ and in TiB_2_ by Nakano et al.^19^. The argument seems only partly consistent with the present results of micropillar compression tests of TiB_2_, in which prism ***a*** slip is observed to operate at room temperature. However, in view of the fact that the CRSS value is much lower for prism ***c*** slip than for prism ***a*** slip in TiB_2_, the argument of the early studies as to which of the two, prism ***a*** slip and basal ***a*** slip, is the primary slip system in transition-metal diborides seems not to make sense at all. The results of micropillar compression tests of ZrB_2_ is also not consistent with the argument of the early studies, since prism ***a*** + ***c*** slip is proved to be only the operative slip system at room temperature. The operation of prism ***a*** + ***c*** slip was also indeed observed in micropillar compression tests of ZrB_2_ by Csanadi et al.^44^. Csanadi et al.^22^ inferred the co-operation of {1$\bar{1}$00}[0001] and {1$\bar{1}$00}<11$\bar{2}$3> slip at room temperature in ZrB_2_ from slip patterns around a cube corner of a nano-indent. Slip on {1$\bar{1}$00}<0001> (prism ***c*** slip) was claimed to operate by Ghosh et al.^21^ for the first time in scratch-induced groove by nano-indentation on a ZrB_2_-SiC composite. However, the operation of {1$\bar{1}$00}[0001] slip was not observed at all in the present micropillar compression tests.

**Figure 6:**
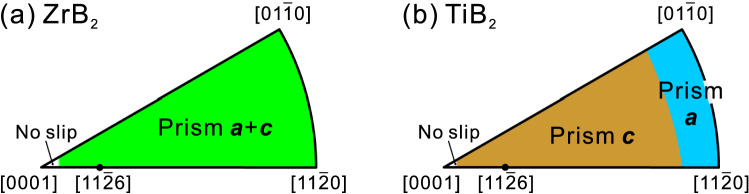
Slip systems predicted to operate depending on crystal orientation for (**a**) ZrB_2_ and (**b**) TiB_2_ at room temperature.

The CRSS values at room temperature are deduced to be 3.01 GPa for {1$\bar{1}$00}<11$\bar{2}$3> slip in ZrB_2_ and 1.72 GPa and 5.17 GPa, respectively for {1$\bar{1}$00}<0001> and {1$\bar{1}$00}<11$\bar{2}$0> slip in TiB_2_. Slip systems expected to operate at room temperature depending on crystal orientation are plotted in Fig. 6a,b, respectively for ZrB_2_ and TiB_2_ with the use of the above CRSS values for the relevant slip systems. Since there is no slip system to operate near the [0001] orientation (Fig. 2d), the approximate corresponding area is left blank to indicate no operative slip system.

**Figure 7:**
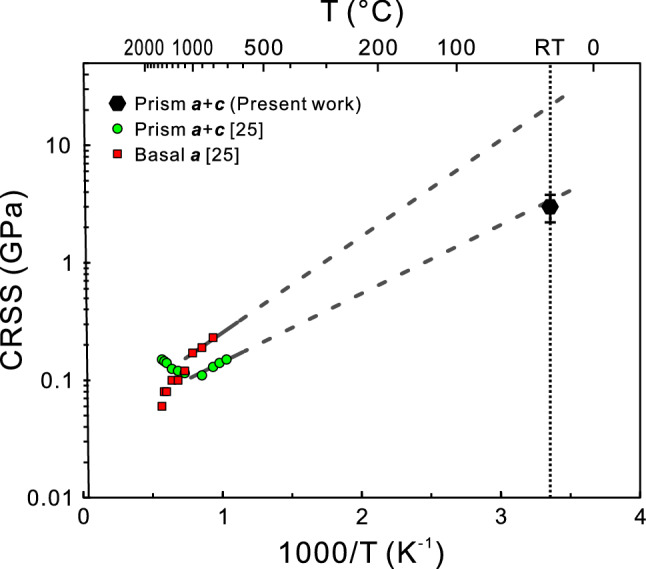
Comparison of room-temperature CRSS values of micropillar specimens and temperature dependent CRSS values of bulk single crystals for {1$\bar{1}$00}<11$\bar{2}$3> slip and (0001)<11$\bar{2}$0> slip in ZrB_2_.

The CRSS value determined for {1$\bar{1}$00} < 11$\bar{2}$3 > slip at room temperature with micropillar specimens are compared with those obtained at high temperatures with bulk single crystals in Fig. 7 for ZrB_2_. Of interest to note in Fig. 7 is that the CRSS value at room temperature coincides well with the extension of the bulk CRSS-temperature relation for prism ***a*** + ***c*** slip in ZrB_2_, indicating the identical deformation mechanism operates at both room temperature and high temperatures. In contrast, the CRSS value for basal ***a*** slip at room temperature might be more than 20 GPa if it is on the extension of the bulk CRSS-temperature relation for basal ***a*** slip in ZrB_2_ (Fig. 7). The expected CRSS value may be too high for basal ***a*** slip to actually operate in ZrB_2_.

### Selection of slip systems and dislocation dissociation modes

**Figure 8:**
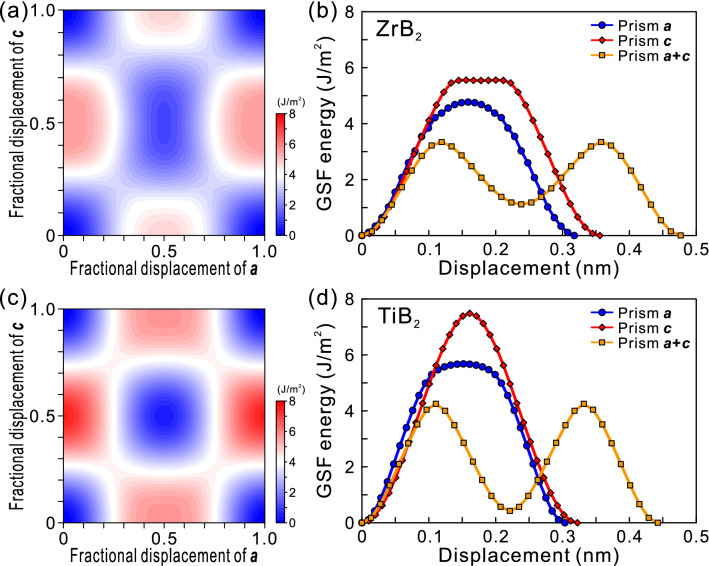
GSF energies on the prism plane in (**a**) ZrB_2_ and (**c**) TiB_2_. The energy landscape along the Burgers vectors of anticipated perfect dislocations for (**b**) ZrB_2_ and (**d**) TiB_2_.

We now discuss some possible reasons about how the operative slip system is selected in micropillar single-crystal specimens at room temperature depending crystal orientation in ZrB_2_ and TiB_2_. In our previous study^25^, we calculated generalized stacking faults (GSF) energies on prism planes of ZrB_2_ and TiB_2_. Figure 8a,c show GSF energies on the prism plane in ZrB_2_ and TiB_2_, respectively. The energy maximum is located at the midpoint along the ***a***- and ***c***-axes, respectively, indicating that the dislocation with the Burgers vector (***b***) of 1/3[11$\bar{2}$0] and [0001] on prism plane may move as a perfect dislocation without dissociation into partial dislocations. On the other hand, an energy minimum is observed to exist at the midpoint along the ***a*** + ***c*** direction, indicating the dislocation with the ***b*** = 1/3[11$\bar{2}$3] on prism plane moves as an extended dislocation with two collinear identical partials separated by a stacking fault, as indicated below.

1 $$1/3[11\bar{2}3] \to 1/6[11\bar{2}3] + {\text{ }}1/6[11\bar{2}3].$$

We indeed observed this dissociation scheme for the ***a*** + ***c*** dislocation on prism plane in ZrB_2_ by TEM in our previous study^25^. The results of our previous GSF calculations are shown in Fig. 8b,d a bit differently from Fig. 14 of our previous paper^25^ to indicate the energy landscape along the Burgers vectors of anticipated perfect dislocations for ZrB_2_ and TiB_2_, respectively. The maximum gradients of the GSF energy curve along the slip direction corresponding to the CRSS values for dislocation motion are tabulated in Table 2. The ease of the operation of slip systems can be evaluated with these values of the maximum gradients of the GSF energy curve (see also for Table 2), as follows.

2 $${\text{Prism}}\,\user2{a} + {\mathbf{c}} < {\text{ Prism}}\,\user2{a} < {\text{ Prism}}\,\user2{c}\quad {\text{for }}\,{\text{ZrB}}_{{\text{2}}} ,$$

3 $${\text{Prism}}\,\user2{a} + {\mathbf{c}} < {\text{ Prism}}\,\user2{c} < {\text{ Prism}}\,\user2{a}\quad {\text{for }}\,{\text{TiB}}_{{\text{2}}} .$$

**Table 2:** The maximum gradients of the GSF energy curve along the slip direction for some slip systems expected to operate and the maximum values of dislocation energy factor for ZrB_2_ and TiB_2_.

	Max. gradient of GSF energy curve (GPa)	Max. value of dislocation energy factor, *Kb*^2^ (GPa m^2^)
**ZrB**_**2**_		
Prism ***a*** + ***c***	41.5	15.5
Prism ***a***	53.2	27.6
Prism ***c***	56.1	31.4
**TiB**_**2**_		
Prism ***a*** + ***c***	54.5	14.9
Prism ***a***	69.1	30.0
Prism ***c***	68.5	27.6

The relative ease for the operation of the slip systems observed for micropillar specimens is correctly predicted with the DFT calculations for ZrB_2_ in that prism ***a*** + **c** slip is the easiest slip system but not completely for TiB_2_ in that the predicted easiest slip system (prism ***a*** + **c** slip) is not actually observed, although the relative ease is correctly predicted for prism ***c*** slip (the easiest system) and prism ***a*** slip. We tried to solve the question as to why prism ***a*** + ***c*** slip is predicted to operate more easily than prism ***c*** slip and prism ***a*** slip in TiB_2_ from the dislocation self-energy view point, since nucleation of mobile dislocations is mandatory for plastic flow to occur for micropillar specimen of brittle material. We calculated the energy factors of anticipated perfect (for prism ***a***, prism ***c*** and basal ***a*** slip) and dissociated (***b*** = 1/2(***a*** + ***c***) for prism ***a*** + ***c*** slip) dislocation for ZrB_2_ and TiB_2_ with the use of elastic constants determined at room temperature^50,51^, and the maximum energy for each dislocation is tabulated in Table 2. However, the above question could not be solved with the order of the magnitude of maximum energy in Table 2.

As described in our previous paper^25^, single crystals of ZrB_2_ and TiB_2_ used in the present study contain some growth faults with varying nature of the growth faults and their density depending on transition-metal diboride. There exists a possibility that these growth faults play a role making the prediction of operative slip systems difficult. More sophisticated work, such as that using growth fault-free single crystals, is definitely needed to answer this question.

## Methods

Single crystals of the two transition-metal diborides, ZrB_2_ and TiB_2_ were grown as previously described in our previous paper^25,52,53^. In brief, single crystals of TiB_2_ and ZrB_2_ were grown with a radio-frequency heated floating-zone furnace from ingots with the stoichiometric compositions, measuring about 1 cm in diameter and 10 cm in length. Single crystals of these transition-metal diborides contain some grown-in defects of varying nature and density depending on diborides (Zr precipitates parallel to basal planes in ZrB_2_ and stacking faults on prism planes in TiB_2_) as detailed in our previous paper^25^. Orientations of single crystals were determined by the X-ray Laue back-reflection method. Four different loading-axis orientations, [0001], [11$\bar{2}$0], [01$\bar{1}$0] and [11$\bar{2}$6], were selected as the compression-axis orientations for ZrB_2_, while two loading-axis orientations, [11$\bar{2}$0] and [11$\bar{2}$6], were selected for TiB_2_. The highest Schmid factors for some possible slip systems ever reported (prism ***a***, prism ***c***, prism ***a*** + **c** and basal ***a***) are listed in Table 1 for each orientation. After mechanical polishing with diamond paste to mirror finish, micropillar specimens with a square cross-section having an edge length *L* ranging from 1.0 to 8.0 μm and an aspect ratio of approximately 1:2.5 were machined from the single crystal with a JEOL JIB-4000 focused ion beam (FIB) apparatus at an operating voltage of 30 kV. A square cross-section was employed to facilitate the identification of slip planes and slip directions. Special care was taken to put one of the possible slip directions (either ***a***, ***c*** or ***a*** + ***c***) on one of the two orthogonal side faces, so that the slip direction is easily determined from the specimen shape change after deformation.

Compression tests were conducted with these micropillar specimens at a nominal strain rate of 1 × 10^−4^ s^−1^ at room temperature using Agilent nanoindenter G200 equipped with a flat punch diamond tip under the displacement-rate-controlled mode. Micropillar specimens were observed before and after deformation by scanning electron microscopy (SEM) with a JEOL JSM-7001FA electron microscope to identify the activated slip plane and slip direction.

## Conclusions

1. Plastic flow is successfully observed in compression at room temperature for both ZrB_2_ and TiB_2_ when tested with small specimens of the micron-meter-size, in spite of the high onset temperatures (700 °C) for plastic flow for bulk single crystals of ZrB_2_ and the absence of plastic flow below 1500 °C for TiB_2_.
2. The operative slip systems identified at room temperature for micropillar specimens are completely different from those identified to operate at high temperatures in the corresponding bulk single crystals for both ZrB_2_ and TiB_2_. {1$\bar{1}$00}<11$\bar{2}$3> slip, which was identified as the primary slip system at high temperatures in bulk single crystals, is only the operative slip system at room temperature in ZrB_2_. While any appreciable plastic flow is not observed in bulk single crystal of TiB_2_ below 1500 °C, {1$\bar{1}$00}<0001> and {1$\bar{1}$00}<11$\bar{2}$0> slip are observed to operate at room temperature in TiB_2_.
3. The CRSS values for all slip systems identified to operate at room temperature in ZrB_2_ and TiB_2_ do not much depend on specimen size (side length, *L*), indicating the high Peierls (frictional) stress for the dislocation motion as often observed in many brittle materials. The bulk CRSS values at room temperature estimated as the value averaged over specimen size are 3.01 GPa for {1$\bar{1}$00}<11$\bar{2}$3> slip in ZrB_2_ and 1.72 GPa and 5.17 GPa, respectively for {1$\bar{1}$00}<0001> and {1$\bar{1}$00}<11$\bar{2}$0> slip in TiB_2_.

## Data Availability

The datasets generated during and/or analysed during the current study are available from the corresponding author on reasonable request.
